# NLRP1 in Cutaneous SCCs: An Example of the Complex Roles of Inflammasomes in Cancer Development

**DOI:** 10.3390/ijms232012308

**Published:** 2022-10-14

**Authors:** Michela Di Filippo, Paulina Hennig, Tugay Karakaya, Marta Slaufova, Hans-Dietmar Beer

**Affiliations:** 1 Department of Dermatology, University Hospital of Zurich, 8091 Zurich, Switzerland; 2 Faculty of Medicine, University of Zurich, 8032 Zurich, Switzerland

**Keywords:** NLRP1, inflammasomes, skin, skin cancer, SCC, inflammation

## Abstract

Protein complexes termed inflammasomes ensure tissue protection from pathogenic and sterile stressors by induction of inflammation. This is mediated by different caspase-1-induced downstream pathways, including activation of the pro-inflammatory cytokines proIL-1β and -18, induction of a lytic type of cell death, and regulation of the release of other pro-inflammatory molecules. Aberrant inflammasome activation underlies the pathology of numerous (auto)inflammatory diseases. Furthermore, inflammasomes support or suppress tumor development in a complex cell-type- and stage-dependent manner. In human keratinocytes and skin, NLRP1 is the central inflammasome sensor activated by cellular perturbation induced, for example, by UVB radiation. UVB represents the main inducer of skin cancer, which is the most common type of malignancy in humans. Recent evidence demonstrates that activation of NLRP1 in human skin supports the development of cutaneous squamous cell carcinomas (cSCCs) by inducing skin inflammation. In contrast, the NLRP1 inflammasome pathway is restrained in established cSCCs, suggesting that, at this stage, the protein complex has a tumor suppressor role. A better understanding of the complex functions of NLRP1 in the development of cSCCs and in general of inflammasomes in cancer might pave the way for novel strategies for cancer prevention and therapy. These strategies might include stage-specific modulation of inflammasome activation or its downstream pathways by mono- or combination therapy.

## 1. Introduction

Inflammation represents a tissue response induced by many different stress factors [1,2]. These include PAMPs (pathogen-associated molecular patterns), highly conserved molecules derived from tissue invading bacteria or viruses, and DAMPs (damage-associated molecular patterns), molecules released by impaired endogenous cells after, for example, trauma or injury. PRRs (pattern recognition receptors) are localized on the surface or inside of the cell and ensure the detection of PAMPs and DAMPs. Immune cells are central for inflammation and are attracted and activated by chemokines and cytokines, which are released by immune or other tissue resident cells upon PRR activation. In most cases, acute inflammation is highly beneficial because it is strictly required for efficient defense against pathogens, repair after injury, or induction of anti-tumor responses. However, when chronic, inflammation can also be detrimental, underlying the pathology of inflammatory diseases and tumor development. There is increasing evidence that inflammation is able to support or inhibit cancer development by acting at all stages of tumorigenesis, namely, tumor initiation, promotion, progression, and metastasis [3,4]. It has been estimated that about 25% of all cancers of epithelial origin might be caused by chronic inflammation, induced by viral or bacterial infection, or associated with other pro-inflammatory conditions [5]. In contrast, there is increasing evidence for efficient T lymphocyte-based immunotherapy against cancer [6]. Indeed, tumor cells proliferate with other cell types in the TME (tumor microenvironment), including mesenchymal cells, such as CAFs (cancer-associated fibroblasts), and different types of immune cells. These immune cells can strongly support cancer development by providing factors for tumor cell proliferation or suppression of anti-tumor immunity [7]. On the other hand, they can also induce anti-tumor immune responses, able to eradicate tumor cells and cure the patient [6]. Therefore, understanding the different, in part opposing, roles of inflammation and immune cells in the cancer development of the individual patient provides the potential for the establishment of efficient treatment options [8].

The IL (interleukin)-1 family consists of 11 members (IL-1β, IL-1α, IL-1Ra, IL-18, IL-36Ra, IL-36α, IL-36β, IL-36γ, IL-37, IL-38, and IL-33) with either pro- or anti-inflammatory activity. IL-1β is a highly conserved, pleiotropic, and very potent pro-inflammatory cytokine that can induce inflammation and, if the dose is high enough, even a septic shock [9]. IL-1β exerts its biological activity by binding to IL-1RI (IL-1 receptor type I), which is ubiquitously expressed. IL-1 activity is controlled by IL-1Ra (IL-1 receptor antagonist), a secreted protein that binds to IL-1RI, but cannot activate the downstream signaling pathway. IL-1 plays important roles in the induction of inflammation, inflammatory diseases, as well as cancer [9,10,11]. IL-1β is initially synthesized as an inactive 31 kDa precursor (proIL-1β), which cannot bind and activate IL-1RI. The cysteine protease caspase-1 is the principal activator of proIL-1β, cleaving the pro sequence off, and thereby generating the active 17 kDa IL-1β [12]. In contrast, proIL-1α neither is a substrate for caspase-1 nor requires proteolytic processing for binding and activation of IL-1RI. Furthermore, caspase-1 is the main activator of proIL-18, which binds and stimulates in its mature form IL-18Rα/β [13]. Unlike IL-1, proIL-18 is ubiquitously expressed and involved in the induction of IFN-γ expression [9].

## 2. Inflammasomes

**Figure 1:**
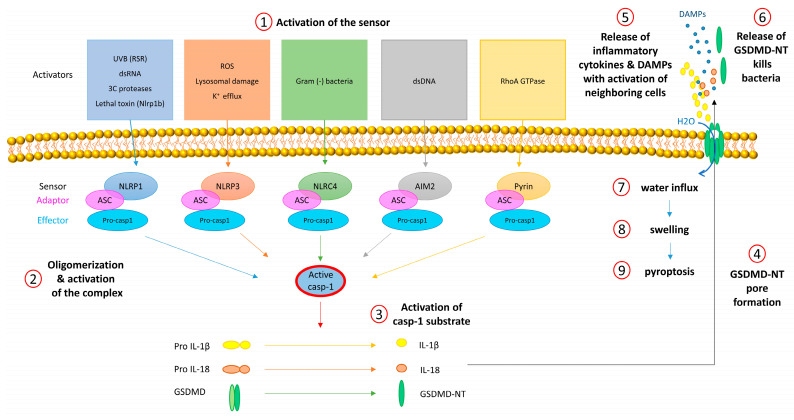
Activation of inflammasomes and downstream mechanisms. (1) Each inflammasome sensor is activated by specific stressors. (2) Activation is associated with oligomerization of the sensor and of the adaptor protein ASC (ASC speck formation) followed by proteolytic self-activation of pro-caspase-1. (3) Active caspase-1 cleaves and thereby activates the pro-inflammatory cytokines proIL-1β and -18 and GSDMD. (4) GSDM-NT oligomerizes and forms pores in the cell membrane. (5) Via GSDMD pores, IL-1β, IL-18, and other molecules are released and induce inflammation by activation of immune and other neighboring cells. (6) Released GSDMD-NT inserts into the membrane of pathogens and kills them. (7) Through GSDMD-NT pores, water enters the cell (8) causing swelling and (9) rupture of the cell. This lytic type of cell death is termed pyroptosis and supports inflammation.

Caspase-1 is expressed as an enzymatically inactive precursor molecule (pro-caspase-1) and its activation occurs upon assembly of inflammasomes [14]. Inflammasomes are multiprotein complexes that contain (i) a sensor protein, such as NLRP1 (NLR (NOD-like receptor) family pyrin domain containing 1), NLRP3, NLRC4 (NLR family CARD (caspase activation recruitment domain) domain containing 4), AIM2 (absent in melanoma 2), or pyrin, which names the corresponding type of inflammasome; (ii) the adaptor protein ASC (apoptosis-associated speck-like protein containing a CARD); and (iii) the effector protein pro-caspase-1 (Figure 1). Inflammasomes have been mainly characterized in immune cells, but are also expressed by keratinocytes [14,15]. They are activated by several different sensor-specific stressors. Once activated, the sensor induces the formation of ASC oligomers, termed ASC specks [16]. Then, pro-caspase-1 is recruited and activated by proximity-induced dimerization [17]. All interactions between the inflammasome sensor, ASC, and pro-caspase-1 are homotypic based on the death domain fold, either the CARD (in ASC, pro-caspase-1, NLRP1, and NLRC4) or the pyrin domain (in ASC, NLRP3, AIM2, and pyrin) [18]. Inflammasomes play key roles in innate immunity protecting from several pathogens [19]. However, their chronic activation, particularly of NLRP3, also underlies numerous common inflammatory diseases, ranging from Alzheimer’s disease, atherosclerosis, and diabetes to rheumatoid arthritis (for cancer, please see 3) [15,20]. As NLRP3 seems to be dispensable for immunity, its targeting represents a promising strategy for the treatment of numerous patients suffering from NLRP3-mediated diseases [21].

In most cases, inflammasome activation requires a preceding priming step for transcriptional induction of expression of proIL-1β, NLRP3, or AIM2 expression and posttranslational modifications of NLRP3 [22,23,24]. This is achieved by LPS-induced TLR (toll-like receptor) 4 activation or stimulation of cells with TNFα, IL-1 itself, or IFN-γ.

Inflammasome activation is often equated with proIL-1β activation. Indeed, owing to its fundamental role in inflammation, inflammatory diseases, and cancer (please, see in 1), proIL-1β is the most important substrate of caspase-1. However, proIL-18 also plays crucial roles in immunity, particularly in the intestine, where it seems to be more important for repair than IL-1 [25,26,27]. Importantly, proIL-18 is not only activated by caspase-1, but also by other proteases [9].

Inflammasome activation in immune cells leads to pyroptosis, a lytic form of cell death that supports inflammation [28]. In 2015, GSDMD (gasdermin D) was identified as a substrate of caspase-1 and the inflammatory caspases -4 and -5 (and caspase-11 in mice) [29,30,31]. Caspase-1 activates GSDMD upon cleavage, inducing oligomerization of its aminoterminal (NT) fragment. Then, the GSDMD-NT oligomer inserts into the cell membrane (and into mitochondrial membranes), creating pores [32,33,34]. Consequently, water molecules can enter the cell, causing its rupture. In this way, pyroptosis induced by GSDMD terminates inflammasome activation. In addition, pyroptotic cells release proinflammatory molecules, including DAMPs, which activate neighboring cells [35]. Moreover, GSDMD-NT can also kill bacteria by membrane insertion, either intracellularly or after GSDMD-NT release by caspase-1 activating cells [33].

Most proteins with extracellular function possess a signal peptide for secretion by the canonical ER (endoplasmic reticulum)/Golgi pathway. However, IL-1α, -β, -18, and other members of the IL-1 family lack such a signal peptide [9]. They are either passively released upon cell lysis and pyroptosis (for instance, IL-1α and IL-33, so-called alarmins [36]) or by different, partially poorly understood pathways, collectively termed unconventional protein secretion [37,38]. Several leaderless proteins, including IL-1α, -β, and -18, are released upon pro-caspase-1 activation by living cells by GSDMD-NT pores [32,33,39]. Therefore, pyroptosis is not a prerequisite for IL-1 release, but the cytokine can be additionally unleashed from different living and non-pyroptotic cell types after GSDMD activation [40,41,42,43,44]. Interestingly, GSDMD pores are dynamically regulated and can be repaired [33,45]. Furthermore, GSDMD is not essential for pyroptosis and IL-1β release because other GSDM family members can compensate for its role [46,47,48].

## 3. Inflammasomes and Cancer

In general, chronic inflammation is believed to support all stages of malignant transformation and represents a hallmark of cancer development [49,50]. In contrast, acute inflammation can induce anti-tumor immunity, causing tumor regression [2,51]. Owing to their central roles in inducing inflammation, inflammasomes are generally considered to be tumor promoters [10,52,53,54,55]. However, this depends on the type of cancer, the type(s) of the activated inflammasome(s) in a given cancer, the cell type(s) where the inflammasome(s) is/are activated, and the time point(s) of activation. 

Polymorphisms of inflammasome genes are associated with different types of cancer [11,54,55]. For example, variants of *AIM2* contribute to the development of cancer of the small intestine and CRC (colorectal cancer) [56,57]. Furthermore, single nucleotide polymorphisms (SNPs) of *NLRP3* are associated with Crohn’s disease, a risk factor for CRC, and a gain-of-function mutation results in poorer survival of patients suffering from CRC and increases the risk of developing melanoma [58,59]. In addition, polymorphisms of *NLRP3*, *proIL-1β*, *caspase-1*, and *IL-1RN* are associated with gastric cancer after *Helicobacter pylori* infection [60,61]. In mice, gastric inflammation and, subsequently, gastric cancer can be induced by overexpression of IL-1β [62]. 

*ProIL-1β* and *IL-1Ra* variants are associated with non-small-cell lung cancer as well [63,64,65,66], and a recent study confirmed the role of IL-1 in the development of lung cancer [67,68]. The Canakinumab anti-inflammatory thrombosis outcome study (CANTOS) aimed to determine whether patients suffering from coronary artery disease profit from canakinumab, a human neutralizing IL-1β antibody [69]. Surprisingly, the successful study also revealed protection from lung cancer [67]. 

IL-1β production in tumors, either by tumor cells themselves or by stromal cells, is associated with a worse prognosis [54,56,70,71,72]. At the molecular and cellular level, IL-1β supports tumor development by different mechanisms. The cytokine directly supports tumor cell proliferation and, consequently, tumor growth [55]. Furthermore, IL-1β recruits MDSCs (myeloid-derived suppressor cells) to the TME and activates them [54]. This heterogeneous population of immature myeloid cells plays a key role in the TME by shifting it to the immunosuppressive side upon suppression of NK (natural killer) cells [53,57] and induction of T_reg_ (regulatory T) cells [73]. Production of IL-1β for MDSCs’ attraction is NLRP3-driven [74] and can induce immunosuppressive CD4^+^ T cell polarization [75], whereas the roles of other types of inflammasomes, such as AIM2, are less well described [76]. It seems that the effects of IL-1β on the inhibition of anti-tumor responses are associated with its chronic activation by inflammasomes [77].

IL-1β, together with IL-18, can also induce epithelial cells to lose their polarity and adherence and to acquire a migratory and mesenchymal cell-like phenotype, a process termed EMT (epithelial-mesenchymal transition) [61]. This is regulated by transcription factors, including SNAIL, and the downregulation of E-cadherin, required for tight junctions. Specifically, IL-1β induces SNAIL and suppresses E-cadherin expression in gastric cancer cells [77] and IL-18 downregulates claudins, which are also tight junction proteins, thereby enhancing breast cancer cell migration [78]. Furthermore, IL-1β enhances the invasiveness of breast ductal cancer cells [79] through induction of MMP9 (matrix metalloproteinase 9) expression, mediated by the transcription factor AP-1 (activator protein 1). 

IL-1, by inducing VEGF (vascular endothelial growth factor) expression, also has a role in angiogenesis [54,61,77,80]. This process is required for small and localized tumors to progress, enlarge, and metastasize [81,82]. IL-1α promotes angiogenesis by induction of VEGF expression and, subsequently, by the VEGFR2 pathway in mice [83]. In a melanoma model, IL-1β expressed by myeloid cells and other pro-inflammatory cytokines induce VEGF expression in endothelial cells, creating a microenvironment favorable for early tumor development [80]. Furthermore, IL-1 alone or via VEGF also regulates later stages of tumor progression, including metastasis [84,85,86].

Currently, several trials are ongoing, studying the potential of canakinumab and anakinra (recombinant IL-1Ra) in mono- and combination therapy for the treatment of patients suffering from different types of cancer [87] and reviewed in [50,87]).

IL-1α also represents a target for cancer therapy [88]. Bermekimap, a human IL-1α neutralizing antibody, was tested in patients suffering from different types of cancer, including advanced colorectal cancer, and reduced cancer-associated cachexia [89,90,91]. 

By the suppression of the tumoricidal function of NK cells through the induction of expression of PD1 (programmed cell death 1), IL-18 can also contribute to tumorigenesis [92].

Pro-tumorigenic roles of IL-1 [11,89] and inflammasomes [91] were also demonstrated in several studies with mice. In a murine model of breast cancer, an anti-PD-1 and anti-IL-1β treatment revealed synergistic effects for enhancing anti-tumor immunity [93].

A comparison of chemical-induced tumor incidence in mice lacking expression of IL-1RI ligands revealed that IL-1Ra expression strongly protects from tumors, IL-1α had a weak protective effect, whereas IL-1β strongly supports tumor development [94]. Similarly, the ablation of NLRP3 expression in mice protects from carcinogenesis and metastasis [95]. In contrast, in a murine tumor model, IL-1β injection resulted in tumor regression [55], demonstrating that IL-1 can also have anti-tumor effects, for example, via induction of Th1/Th17 responses [10,54]. Similarly, the inflammasome pathway seems to have a protective role in murine colitis-associated cancer models [53,96]. In DSS (dextran sulfate sodium)- and AOM (azoxymethane)-induced colitis models, ablation of NLRP3 expression increases the number of intestinal tumors [25,26,97]. At least in these models, IL-18 rather than IL-1 seems to have a key protective role, as it supports the repair of the colonic epithelium after injury [25,26,98]. Experiments with mice lacking ASC, caspase-1, and IL-18 expression suggest that IL-18 is required for normal intestinal microbiota [99]. Furthermore, the NLRC4 inflammasome contributes to inflammation-induced colon cancer [100].

Other members of the IL-1 family can have roles in tumorigenesis, too. It is believed that IL-33 is biologically active in its pro-form [101], although more potent when cleaved [102], and has pro-tumorigenic activity [11,87,88], particularly at early stages of cancer development [103,104]. ProIL-36, on the other hand, requires proteolytic activation by different proteases in order to be active and is associated with the inhibition of tumor development [76,77].

Finally, inflammasome proteins have, at least in part, inflammasome-independent roles relevant for cancer development, adding another level of complexity. This is especially relevant for ASC. ASC was originally identified as TMS1 (target of methylation-induced silencing-1), whose expression is suppressed in different types of cancer [105,106]. ASC is not only essential for inflammasome activation, but can also interact with the pro-apoptotic Bcl-2 family member Bax, translocates to mitochondria, and induces apoptosis via the release of cytochrome C [79]. Therefore, ASC is not only an inflammasome adaptor, but also a pro-apoptotic tumor suppressor, independent of the inflammasome pathway [106]. Therefore, it might be difficult to estimate which pathway is more relevant when ASC expression is silenced in cancer.

## 4. The NLRP1 Inflammasome

When NLRP1 was cloned for the first time, it was believed that the protein played a role in apoptosis [80,81]. Later, NLRP1 was the first inflammasome sensor identified [82]. Although originally characterized in the monocytic cell line THP-1 [82], NLRP1 is expressed at particularly high levels in epithelial cells, such as epidermal keratinocytes, and in the brain [83].

**Figure 2:**
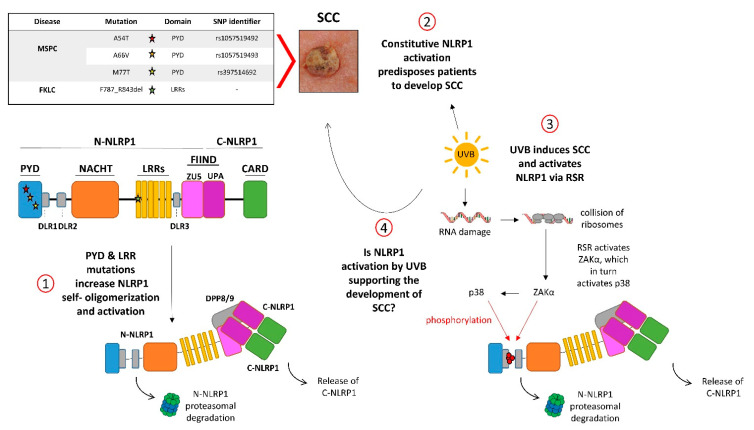
NLRP1, UVB, and skin cancer. (1) NLRP1 is a five-domain self-processing protein. Self-processing occurs in the FIIND (function-to-find domain) between the ZU5 and UPA subdomains, generating the inhibitory N-NLRP1 and the effector C-NLRP1. Germline gain-of-function mutations of *NLRP1* (stars) cause the inflammatory skin syndromes MSPC (multiple self-healing palmoplantar carcinoma) and FKLC (familial keratosis lichenoides chronica), predisposing patients to develop SCCs (2). These mutations induce self-oligomerization and -activation of NLRP1 by release from C-NLRP1 from the dipeptidyl peptidases DPP8/9 and the inhibitory N-NLPR1 (1). (3) The UVB spectrum of the sunlight activates NLRP1 via the ribotoxic stress response (RSR) upon sensing of collision of ribosomes by the kinase ZAKα. Then, ZAKα phosphorylates and activates the stress-induced kinase p38, and both enzymes phosphorylate N-NLRP1 in DLR1 (disordered linker region 1), causing its proteasomal degradation and release of C-NLRP1. (4) It is tempting to speculate that NLRP1 activation either by UVB or by MSPC-causing mutations induces the development of SCCs by overlapping mechanisms.

The NLRP1 protein consists of 1473 amino acids and five different domains [107]: an aminoterminal PYD (pyrin domain), followed by a NACHT domain, six leucine-rich repeats (LRRs), a FIIND (function-to-find domain), and a carboxyterminal CARD (Figure 2). The FIIND, consisting of ZU5 (ZO-1 and UNC5) and UPA (UNC5, PIDD, and Ankyrins) subdomains, is a constitutive proteolytic self-activation domain with cleavage occurring at Phe1212 at the border between ZU5 and UPA [84,85]. The carboxyterminal part (NLRP1-CT) represents the effector fragment, but remains inhibited upon interaction with the aminoterminal inhibitory fragment (NLRP1-NT). In most cases, activation of NLRP1-CT is induced by proteasomal degradation of NLRP1-NT [86].

NLRP1 is the central inflammasome sensor in human skin and is expressed by human keratinocytes [108]. In addition, human keratinocytes can express and activate the AIM2 inflammasome, which might underlie skin inflammation in psoriasis, as well as the NLRP3 inflammasome [109,110]. Human keratinocytes in vitro express all NLRP1 inflammasome proteins, including proIL-1α, -β, and -18 [111], and irradiation of the cells with a physiological dose of UVB induces NLRP1 inflammasome activation [112], which is believed to underlie the induction of sunburn [62]. UVB-induced NLRP1 activation requires the activity of the stress-induced protein kinases p38 and JNK [113]. Recently, it was shown that this is induced by the kinase ZAKα (leucine-zipper and sterile-alpha motif kinase) [114,115] upon activation of the ribotoxic stress response (RSR) pathway [116] (Figure 2). The RSR is induced by the collision of ribosomes upon UVB radiation and sensed by ZAKα binding directly to the ribosomes. Then, ZAKα is activated, inducing phosphorylation of p38 and JNK. Finally, activated ZAKα and p38 directly activate NLRP1 by phosphorylation [114,115]. In non-stressed cells, NLRP1 is inhibited upon binding to DPP8 (dipeptidyl peptidase 8) and 9, partially via interaction of the aminoterminus of NLRP1-CT with the active site of the peptidases [117,118]. Consequently, treatment of cells with the anti-cancer drug and DPP8/9 active site inhibitor talabostat (Val-boroPro, PT-100) [119] induces NLRP1 activation in human keratinocytes and the downstream secretion of high levels of IL-1β [48,120]. It is not yet known whether long-term treatment of patients with talabostat induces SCCs, because the drug is not yet approved for the treatment of solid cancers (clinical trial NCT04171219 is ongoing). Furthermore, human NLRP1 can be activated by dsRNA [121] and viral 3C proteases [122,123]. Both stimuli result in the degradation of NLRP1-NT and the release of the effector NLRP1-CT [111]. 

A similar mechanism of activation was identified previously for murine Nlrp1b. In contrast to humans, three different Nlrp1 paralogues are expressed in mice, with Nlrp1b possessing the strongest homology to human NLRP1 [83]. Upon infection with *Bacillus anthracis*, Nlrp1b but not human NLRP1 is proteolytically activated in the aminoterminal region by the lethal factor, a protease of anthrax lethal toxin [121,124]. Then, the shortened aminoterminal fragment of Nlrp1b is ubiquitinated and degraded by the proteasome, finally causing Nlrp1b activation [122,123]. This mechanism is termed functional degradation [122].

However, the roles of human NLRP1 are only poorly conserved between humans and mice, demonstrating that the NLRP1 is a relatively young evolutionary pathway [101]. Mechanistically, only human NLRP1 but not murine Nlrp1b is activated by dsRNA, ORF45, UVB, and the ribotoxic stress response [102,114,115,125]. Talabostat also activates Nlrp1b in murine immune cells; however, this occurs independently of ASC expression and induces mainly pyroptosis with very low levels of secreted IL-1β [126,127]. Furthermore, expression of Nlrp1b in murine keratinocytes is very low and, although sunburn in mice is IL-1β- and caspase-1-dependent, this is not mediated by keratinocytes, but most likely by a currently unknown immune cell type [128].

Single nucleotide polymorphisms (SNPs) of the *NLRP1* gene are associated with different (auto)inflammatory diseases, which mainly affect the skin, including vitiligo, Addison’s disease, and NAIAD (NLRP1-associated autoinflammation with arthritis and dyskeratosis) [101,129,130,131]. Furthermore, germline gain-of-function mutations of *NLRP1* cause two rare inflammatory skin diseases, MSPC (multiple self-healing palmoplantar carcinoma) and FKLC (familial keratosis lichenoides chronica) [108]. These discoveries demonstrate that NLRP1 plays a particularly important role in skin inflammation. Most importantly, patients with these mutations are predisposed to develop cutaneous SCCs, demonstrating a link between NLRP1-induced inflammation and cancer development in human skin [108] (Figure 2).

## 5. Inflammasomes in Skin Cancer and NLRP1 in Cutaneous SCCs

Cutaneous SCCs and BCCs (basal cell carcinomas) originate from keratinocytes and represent the main types (SCC: 20–30%, BCC: 70–80%) of NMSC (non-melanoma skin cancer), the most prevalent cancer worldwide with further increasing incidence rates [132]. Nearly 90% of NMSC is caused by exposure to UV radiation, either from sunlight exposure or from tanning beds [133]. Indeed, UVA and particularly UVB can cause skin cancer by inducing DNA damage, inflammation, and immune suppression [134]. In addition to UV, immunosuppression, infection by human papillomavirus (HPV), light skin, old age, exposure to organic chemicals and ionizing radiation, and genetic predisposition represent other risk factors [135,136]. Even though invasion and metastasis are rare events in SCC and BCC patients (for SCCs, about 5%, and for BCCs, 0.0028–0.55%), the high number of patients suffering from them creates a significant burden for the public health system [137,138,139]. Excisional surgery is the most efficient gold standard therapy, but it also represents a frequent cosmetic issue, because BCCs and SCCs develop mainly on the sun-exposed body surface, such as the face. 

It is believed that short-term activation of NLRP1 in keratinocytes by UVB with the subsequent release of IL-1 underlies sunburn in humans [62,112,114]. In contrast, chronic NLRP1 activation is responsible for persistent inflammation, which can be considered as tumor promoter in the skin [3,4], which might be mediated by inflammation-associated ROS and IL-1β (Figure 2). Consistently, patients with germline gain-of-function mutations of *NLRP1* suffer from chronic inflammation of the skin and have a high risk of developing cutaneous SCCs [108]. However, the expressions of ASC [140], NLRP1, pro-caspase-1, and proIL-1β are suppressed in established human cutaneous SCC tumors and cell lines, most likely by the promoter methylation [141]. Silencing of ASC expression has been demonstrated in several types of cancer [106,142,143]. It is not known whether SCC cells profit from ASC suppression owing to the pro-apoptotic function of the protein or to its essential role in inflammasome activation. However, reduced expression of the other inflammasome components suggests that inflammasome suppression could have a role in cancer progression and is more than just a bystander effect [141]. Furthermore, expression of AIM2 is increased in human SCCs [144] and SCC cell lines [141] and supports the growth and invasion of cutaneous carcinomas [144].

The NLRP3 inflammasome contributes to the development of other types of human carcinomas [52]. In oropharyngeal SCCs, expression of NLRP3, ASC, caspase-1, and proIL-1β/-18 is increased, suggesting a role in tumor development [145]. In related head and neck SCCs, the NLRP3 inflammasome supports tumorigenesis, survival, and invasiveness [146,147]. Furthermore, NLRP3 supports the resistance of oral SCCs to 5-fluorouracil in vivo and in vitro [148]. NLRP3 expression is also increased in human BCCs [149] and SCC cell lines [141].

Evidence for the roles of inflammasomes in skin cancer development also comes from mouse models. Experiments based on chemically induced skin carcinogenesis revealed an important role for IL-1α derived from keratinocytes/tumor cells [150]. IL-1α induces NF-κB activation in an autocrine manner, which in turn prevents the expression of differentiation markers. Furthermore, IL-1α signaling is required for pro-inflammatory gene expression [150]. It is noteworthy that, in contrast to human keratinocytes, which express proIL-1α and -β, murine keratinocytes express mainly proIL-1α [128]. In the DMBA/TPA model of skin carcinogenesis, expression of IL-1RI, caspase-1, NLRP3, and ASC—the latter in myeloid cells—supports the incidence of papillomas [151,152]. However, specific deletion of ASC expression in keratinocytes results in more skin lesions, most likely due to an additional pro-apoptotic role of ASC independent of the inflammasome pathway in keratinocytes [106,152]. Using the same model, Gasparoto et al. reported increased papilloma incidence and volume in mice lacking expression of ASC and caspase-1, suggesting that the inflammasome is required for protective immune responses [153]. It might be that different housing conditions of mice are responsible for these contradictory results. Furthermore, the IL-1 family member IL-33 expressed by tumor cells supports skin cancer development in mice via an IL-33/TGF-β feedforward loop [103].

UV is also the most important risk factor for melanoma, which derive from melanocytes and represent a very dangerous type of skin cancer [154]. SNPs of *NLRP3* and particularly of *NLRP1* are associated with the development of melanoma [59]. As in other types of human cancer, the expression of ASC is downregulated by promoter hypermethylation in melanoma [142]. However, depending on the stage, ASC has different roles in human melanoma: it acts as a tumor suppressor in primary tumors, but as a tumor promoter in metastatic melanoma, the latter via inflammasome-mediated IL-1β secretion [143]. Furthermore, it was suggested that expression and activation of the NLRP3 inflammasome in human melanoma cells correlate with malignancy and with spontaneous IL-1β secretion by late-stage melanoma [155]. Later, it was demonstrated that NLRP1 rather than NLRP3 promotes melanoma growth and suppresses apoptosis [156]. In mice, the NLRP3 inflammasome, IL-1β, and IL-18 support melanoma growth, migration, and metastasis [92,95,157,158].

## 6. Conclusions and Outlook

It is well accepted that inflammasomes play key roles in cancer development. However, IL-1 and other inflammasome effector pathways seem to have distinct and sometimes even opposite functions in carcinogenesis, dependent on the relevant types of inflammasomes and effector pathways, cell types that express and activate these inflammasomes, and the time points at which the activation occurs. In general, IL-1 and inflammasomes are considered tumor promoters, particularly at the early stages of cancer development [50]. Consequently, inhibition of IL-1 by anakinra or canakinumab [50] or of the NLRP3 inflammasome [21] might prevent cancer development or have therapeutic efficacy at the beginning of tumor development, as demonstrated by the CANTOS trial [67,68]. However, in most cases, cancer is diagnosed not until it causes discomfort, which occurs when cancer has already progressed. At this later stage, IL-1 and inflammasome are often considered to be tumor suppressors and several clinical studies for the treatment of cancer patients by either IL-1 mono- or combination therapy with, for example, immune checkpoint inhibitors [50,87,93] are ongoing.

Although inflammasome activation is believed to be mainly associated with immune cells, the NLRP1 inflammasome is highly expressed by epidermal keratinocytes rather than by immune cells [101]. The fact that patients with germline gain-of-function mutations of *NLRP1* are predisposed to develop cSCCs proves that the NLRP1 inflammasome represents a tumor promoter pathway in the development of NMSC [108]. Most likely, this is mediated by NLRP1 inflammasome activation in keratinocytes, IL-1-induced skin inflammation, and possibly IL-1-dependent immunosuppression. Anti-cancer immunity plays a key role in keeping the development of cSCCs under control, as immunosuppression of organ transplant recipients predisposes them to cSCCs [159]. Similarly, UVB induces inflammation (sunburn), which is likely mediated by NLRP1 inflammasome activation in keratinocytes, as well as the development of skin cancer [62,111,112]. Furthermore, it is well known that UVB induces immunosuppression in the skin by different mechanisms [134]. Therefore, it is tempting to speculate that NLRP1-dependent IL-1 production by keratinocytes, when this occurs regularly or even chronically, contributes to keratinocyte proliferation and immunosuppression, supporting the early stages of cSCC development.

Nevertheless, the expression of not only ASC [140] but of all inflammasome proteins is suppressed in established cSCC, suggesting a tumor suppressor role of the NLRP1 inflammasome pathway during cSCCs progression [141]. As the NLRP1 pathway is not conserved in the skin of mice, to prove this hypothesis and to elucidate the underlying molecular mechanisms in vivo will be difficult [128].

In conclusion, the NLRP1 inflammasome seems to have opposing roles in the early versus late development of cSCCs. IL-1 blockade or NLRP1 inhibition in the skin might be protective and antagonize the early development of cSCCs. This option might be particularly useful for patients suffering from MSPC and FKLC when they start developing skin lesions as a consequence of NLRP1 activation. Indeed, as NLRP1 is expressed at particularly high levels by keratinocytes in human skin, its pharmacological targeting could be achieved by topical treatment, thus without major side effects.

In contrast, NLRP1 or IL-1RI activation could represent useful novel strategies for patients suffering from advanced cSCCs. Indeed, the TLR agonist Aldara^TM^, which is also an activator of NLRP1, is already used for patients suffering from BCCs [160].
